# The Interplay Between Respiratory Microbiota and Innate Immunity in Flavor E-Cigarette Vaping Induced Lung Dysfunction

**DOI:** 10.3389/fmicb.2020.589501

**Published:** 2020-12-16

**Authors:** Zahira Quinones Tavarez, Dongmei Li, Daniel P. Croft, Steven R. Gill, Deborah J. Ossip, Irfan Rahman

**Affiliations:** ^1^Department of Clinical and Translational Research, University of Rochester Medical Center, Rochester, NY, United States; ^2^Department of Medicine, Pulmonary Diseases and Critical Care, University of Rochester, Rochester, NY, United States; ^3^Department of Microbiology and Immunology, University of Rochester Medical Center, Rochester, NY, United States; ^4^Department of Public Health Sciences, University of Rochester Medical Center, Rochester, NY, United States; ^5^Department of Environmental Medicine, University of Rochester Medical Center, Rochester, NY, United States

**Keywords:** e-cigarettes, vaping, microbiota, host-microbiota interaction, innate immunity, lung injury

## Abstract

Global usage of electronic nicotine delivery systems (ENDS) has been increasing in the last decade. ENDS are non-combustible tobacco products that heat and aerosolize a liquid containing humectants, with added flavorings and often nicotine. Though ENDS are promoted as a less harmful alternative to smoking, current evidence links their use to a wide range of deleterious health effects including acute and chronic lung damage. ENDS can elicit an inflammatory response and impair the innate immune response in the lungs. Exposure to ENDS flavorings results in abnormal activation of the lung epithelial cells and β-defensins, dysfunction of the macrophage phagocytic activity, increased levels of mucin (MUC5AC) and abnormal activation of the neutrophilic response (NETosis). ENDS menthol flavorings disrupt innate immunity and might be associated with allergies and asthma through activation of transient receptor potential ankyrin 1 (TRAP1). Recent studies have expanded our understanding of the relationship between the homeostasis of lung innate immunity and the immunomodulatory effect of the host-microbiota interaction. Alterations of the normal respiratory microbiota have been associated with chronic obstructive pulmonary disease (COPD), asthma, atopy and cystic fibrosis complications which are strongly associated with smoking and potentially with ENDS use. Little is known about the short-and long-term effects of ENDS on the respiratory microbiota, their impact on the innate immune response and their link to pulmonary health and disease. Here we review the interaction between the innate immune system and the respiratory microbiota in the pathogenesis of ENDS-induced pulmonary dysfunction and identify future areas of research.

## Introduction

Global usage of electronic nicotine delivery systems (ENDS) has increased in the last decade, especially among youth and young adults (Besaratinia and Tommasi, 2019). The prevalence of ENDS among middle- and high-school students in the United States was 10.5 and 27.5%, respectively, in 2019 (Cullen et al., 2019). ENDS are non-combustible tobacco products that heat and aerosolize a liquid containing humectants and solvents, mainly propylene glycol (PG) with or without vegetable glycerin (VG), with added flavorings and often nicotine (Cheng, 2014; Beauval et al., 2017). Though ENDS are promoted as a less harmful alternative to smoking, current evidence links their use to a wide range of deleterious health effects. A growing body of evidence supports the association between ENDS use and addiction (Primack et al., 2018), cardiovascular disease (D’Amario et al., 2019; MacDonald and Middlekauff, 2019), cancer (Wu et al., 2020), and respiratory disease, including acute, and chronic lung damage (Gotts et al., 2019).

The recent outbreak of electronic cigarettes, or vaping, product use-associated lung injury (EVALI) raised national concern about the harmful effects of ENDS products and constituents (Krishnasamy, 2020). Similar to smoking, ENDS flavoring chemicals can elicit an inflammatory response in the lungs and might be associated with asthma, chronic obstructive pulmonary disease (COPD) and adult respiratory disease syndrome (Gerloff et al., 2017). In addition, the risk of wheezing and other respiratory symptoms is greater in ENDS users as compared to non-users and lower when contrasted to smokers (Li et al., 2019; Xie et al., 2020). Moreover, evidence suggests that ENDS flavorings impair the innate immune response in the lungs (Clapp et al., 2017; Gerloff et al., 2017).

The respiratory microbiota has also shown to play a crucial role in the lung innate immunity and inflammatory response in health and disease (Segal et al., 2016; Dickson et al., 2018). Recent pivotal studies have expanded our understanding of the relationship between the homeostasis of lung innate immunity and the immunomodulatory effect of the host-microbiota interaction. In addition, previous reports have linked the respiratory microbiota to the inflammatory response against environmental exposures such as smoking (Jaspers, 2014) and household coal burning products (Hosgood et al., 2014). Further, early studies are reporting that flavors might enhance the antibacterial activity from ENDS liquids and might potentially induce microbial dysbiosis in the airways (Fuochi et al., 2020), disrupting the immunomodulatory effect of the microbiota. Thus, existing, and emerging evidence suggest a relationship among exposure to ENDS flavorings, changes in the respiratory microbiota, and lung disease, specifically the pathogenesis of flavor vaping induced lung dysfunction. Here we will discuss the associated innate-immune mechanisms in relation with the respiratory microbiota in the pathogenesis of ENDS-induced lung dysfunction and identify future areas of research.

## The Respiratory Microbiota Shapes Lung Innate Immune Responses

Since the first published report challenging the lung sterility paradigm (Hilty et al., 2010), researchers’ attention has focused on the investigation of the respiratory microbiota, i.e., the commensal respiratory microbial community in the human airways (Charlson et al., 2011; Beck et al., 2012; Pezzulo et al., 2013). The respiratory tract harbors a low biomass, low diversity bacterial community that play an important role on maintaining lung homeostasis and as part of the local innate immune response (O’Dwyer et al., 2016). In healthy individuals, members of the respiratory microbiota are cell-associated (Dickson et al., 2014) and correspond to four bacteria phyla: Bacteroidetes (including *Prevotella* spp.), Firmicutes (including *Streptococcus* spp. and *Veillonella* spp.), Proteobacteria, and Actinobacteria (Segal et al., 2016; Dickson et al., 2017), and the less studied fungi (*Eremothecium*, *Systenostrema*, and *Malassezia)* and viral communities (*Anelloviridae* family) (Cui et al., 2015; Young et al., 2015).

### The Airways Microbiota and the Oral Microbiota Share Similar Bacterial Membership in Healthy Individuals

The microbial community membership in the respiratory tract reflects the net effect of three factors: (a) microbial immigration from the oropharynx (micro-aspiration or inhalation) (Bassis et al., 2015; Dickson and Huffnagle, 2015); (b) microbial elimination through innate and adaptive immune responses (cough reflex, mucociliary clearance) (Dickson and Huffnagle, 2015); and, (c) local growth conditions at specific sites, e.g., temperature, pH, partial pressure of oxygen, and nutrient availability (Dickson and Huffnagle, 2015; Dickson et al., 2018). Initial studies reported differences in the microbiota throughout the upper respiratory tract (URT) and the lower respiratory tract (LRT) in healthy individuals; however, recent reports have shown that bacterial members of the URT and LRT are similar to those from the oral microbiota (Dickson et al., 2017), except by the presence of *Tropheryma whipplei* in the LRT (Beck et al., 2015) and the higher microbial density in the URT (Charlson et al., 2011). A unified airway theory developed to explain the similarities observed between the microbiota in the upper and lower airways and how this relates to respiratory diseases including COPD, cystic fibrosis, and asthma (Hanshew et al., 2017).

### The Crosstalk Between the URT Microbiota and the Host-Immune Interface Depends on the Airway’s Epithelium Integrity

The airways epithelium provides a biophysical protective barrier (Parker and Prince, 2011; Galeas-Pena et al., 2019) and a site of interaction with the respiratory microbiota. The respiratory microbiota have been shown to influence the lung architecture (Yun et al., 2014) and respiratory system maturation and development (Man et al., 2017). The complex and dynamic respiratory microbiota colonizes the epithelial surface area (Boyton et al., 2013) and plays a crucial role in orchestrating innate and adaptive immune responses (Wu and Segal, 2018) against pathogenic microorganisms, pollutants, toxins, or nanoparticles (Mathieu et al., 2018; Wu and Segal, 2018; Invernizzi et al., 2020). The epithelium barrier provides a checkpoint to balance immunomodulatory actions and proinflammatory activities, and to regulate tissue remodeling. In conditions such as COPD and asthma, epithelial dysfunction might dysregulate the immunological response to the respiratory microbiota and trigger a chronic inflammation (Yang et al., 2020). Further research is needed to understand the mechanisms involved in the two-way host-microbiota interaction; however, the airways’ epithelial integrity maintained by the tight junctions-constitutes a critical factor in the host-microbiota interplay and in the lung innate immune and inflammatory responses (Schneeberger and Lynch, 1992; Macia et al., 2012).

### The Respiratory Microbiota Promotes an Immunotolerant Environment in the Respiratory Tract

According to Sommariva et al. (2020), the respiratory microbiota promotes an “immunotolerant environment” in the respiratory tract by interacting with the resident and recruited immune cells and controlling the inflammatory responses at the respiratory air-liquid interface (Segal et al., 2016). The pattern recognition receptors (PRRs) located in the airway epithelium, dendritic cells and alveolar macrophages recognize commensal and pathogenic microbial molecules and differentiate “danger” and “safe” signals through (1) cooperatively interacting with other receptors and, (2) eliciting downstream signaling mechanisms to release cytokines and chemokines (Matzinger and Kamala, 2011; Swiatczak and Cohen, 2015). Furthermore, repeated exposure to pathogen-associated molecular patterns and damage-associated molecular patterns from members of the respiratory microbiota induces PRRs tolerance, through the toll-like receptors in the dendritic cells and alveolar macrophages (Butcher et al., 2018; Zeng and Jewell, 2019). Moreover, disturbance of the airways microbiota might exacerbate inflammation and impair phagocytic activity (Man et al., 2017). For instance, Dicker et al. (2018) showed that neutrophil extracellular trap (NET) complexes were correlated with low microbial diversity, impaired neutrophil phagocytic activity, and higher abundance of *Haemophilus* species in patients diagnosed with COPD. Thus, the respiratory microbiota interacts with the airway epithelium and phagocytic cells in a positive feedback loop to develop immunological tolerance and prevent exaggerated inflammatory responses. Further research is needed to elucidate specific mechanisms explaining immunological tolerance in the respiratory tract and to identify potential intervention targets in respiratory pathologies.

### The Respiratory Microbiota Regulates the Airway Mucus and Antimicrobials Production

The production of mucus and antimicrobial peptides and proteins is a key element of the innate immune system of the lung. The airway surface liquid facilitates the mucociliary transport of toxicants, microorganisms, or particulates; lubricates and hydrates the airways; and, contributes to the epithelial barrier (Widdicombe, 2002b). The mucus is produced by the respiratory tract secretory cells (tracheobronchial submucosal glands, Goblet cells, and Club cells) and is composed of water, mucins, salts and other macromolecules (Marriott, 1990; Adler et al., 2013); the role of the pulmonary ionocytes is still unknown (Montoro et al., 2018). MUC5AC and MUC5B are the major glycoprotein components in the mucus (Gum, 1992) and determine its functionality in healthy individuals and asthmatic patients (Welsh et al., 2017). The respiratory microbiota shapes the production of mucus by the airway epithelium, though the mechanisms are still unknown (Yun et al., 2014). Remot et al. (2017) used a pathogen-free mice model to show that the respiratory microbiota increased the production of MUC5AC in response to household dust mites.

Other secreted protective mediators in the airway surface liquid include lysozyme, lactoferrin, lipocalins, peroxidase, aminopeptidases, secretory phospholipase A_2_, immunoglobulin A, lung, and nasal epithelium clone protein, collectins (surfactant protein A and surfactant protein D), mannan-binding lectin, cathelicidins, and β-defensins (Singh et al., 1998; Diamond et al., 2000; Schutte and McCrayJr., 2002; Fujita et al., 2004; Dürr et al., 2006; Haczku, 2008; Kawamoto et al., 2014; Hiemstra et al., 2015; Huff et al., 2019). β-defensins and cathelicidins are endogenous host defense peptides secreted by the airway epithelium (Dürr et al., 2006; Tecle et al., 2010); both show antimicrobial and immunomodulatory effects and are involved in shaping the microbiota composition (van der Does et al., 2018). For instance, Jones et al. (2014) showed β-defensins shape the nasopharyngeal microbiota composition increasing the risk of otitis media in children. Several mechanisms have been implicated in the regulation of the microbiota composition by β-defensins, including direct antimicrobial activity (Bakshani et al., 2018) and immunomodulation of inflammatory responses triggered by members of the microbiota (Wassing et al., 2015).

## ENDS Flavorings Disrupt the Lung Innate Immunity

ENDS flavorings constitute the molecules involved in the perception of flavor. In contrast, flavors refer to the experience of taste and smell of e-liquids (Allen et al., 2016). Flavors are associated with ENDS experimentation and initiation in youth, continued use in users of all ages, and underestimation of the negative effects on health (Romijnders et al., 2018; Cullen et al., 2019; Schneller et al., 2019). More than 8,000 flavors are available on the current market with some of them mimicking combustible tobacco products (menthol or tobacco), fruits (berries, citrus, tropical), dessert (coconut, cake, butter, banana, cookie, etc.), alcohol (champagne, mojito, vodka, rum, piña colada), sweets (caramel, chocolate, vanilla), candy (cotton candy, bubble gum), among others (Krüsemann et al., 2019). Moreover, ENDS flavors enhance exposure to nicotine among users by soothing the bitterness and harshness effects of nicotine, and inducing and maintaining addiction (Ha et al., 2015; Kim et al., 2016). Flavoring additives in e-liquids are derived from the food industry, and are generally recognized as safe (GRAS) only for oral ingestion, and include benzaldehyde, cinnamaldehyde, diacetyl, ortho-vanillin, coumarin, pentanedione, acetoin, maltol, eucalyptol, ethyl vanillin, dl-menthol, and flavoring enhancers (Allen et al., 2016; Muthumalage et al., 2017; Czoli et al., 2019; Table 1). These flavoring chemicals have potential to induce oxidative stress and inflammatory responses when inhaled.

**TABLE 1:** Summary of E-liquid flavorings effects on the lung innate immunity and the microbiota.

E-liquid flavor wheel (Krüsemann et al., 2019)	Flavor	Flavoring chemicals in e-liquids	Effects	References
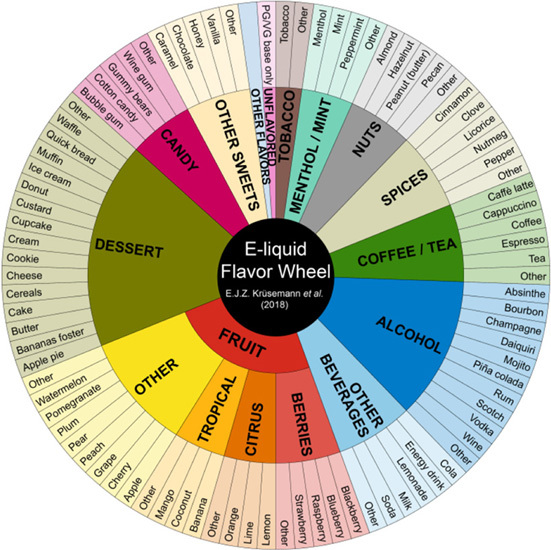
	Cinnamon	Benzaldehyde Cinnamaldehyde Ethyl vanillin Cinnamic acid Methyl eugenol	Cinnamaldehyde suppressed IL-8 secretion in HBEs and human lung fibroblasts and decreased transepithelial resistance in HBEs (lost of AEC integrity).	Gerloff et al., 2017
			Cinnamaldehyde reduced phagocytic activity and cell viability among AMs, PMNs and NK; induced abnormal NET activation and inflammation	Clapp et al., 2017
			Cinnamaldehyde dysregulated mucociliary clearance increasing susceptibility to infection	Clapp et al., 2019
	Tobacco	Butanoic acid 3-(1-Methyl-2-pyrrolidinyl)pyridine β-Nicotyrine 3-methyl-1-phenyl-1H-pyrazole	Tobacco flavored e-liquids induced autophagy of human middle ear epithelial cells and increased mucin production	Go et al., 2020
	Sweet (vanillin)	Ethyl maltol Piperonal Vanillin Isobutyl caproate	Vanillin induced IL-8 secretion in HBEs and human lung fibroblasts and with no effect on the AEC barrier function	Gerloff et al., 2017
	Menthol	Pyrazine,2,3,5-Trimethyl γ-Octalactone dl-Menthol δ-Decalactone	Mentholation in cigarettes decreases the bacterial diversity and presence of human pathogens	Chopyk et al., 2017
			Menthol flavored e-liquids induced autophagy of human middle ear epithelial cells and increased mucin production	Go et al., 2020
			Menthol might induce neurogenic inflammation through activation of TRAP1 receptors in asthma	Yang and Li, 2016
			Menthol has antimicrobial activity against *Staphylococcus aureus* and *Streptococcus epidermidis*	Odeyemi and Oluwajoba, 2011

*Table 1 shows a figure depicting the E-liquid flavor wheel. This figure appears in a publication in the Nicotine and Tobacco Research Journal. The paper provides the following information: ©The Author(s) 2018. Published by Oxford University Press on behalf of the Society for Research on Nicotine and Tobacco. This is an Open Access article distributed under the terms of the Creative Commons Attribution License (http://creativecommons.org/licenses/by/4.0/), which permits unrestricted reuse, distribution, and reproduction in any medium, provided the original work is properly cited.*

### Immunosuppressive Effects of Nicotine on the Respiratory Tract

Nicotine has been shown to increase mucin production and mucus viscosity through activation of the α_7_-nAChR (nicotinic acetylcholine receptor) in the airways (Gundavarapu et al., 2012). Nicotine might reduce allergen-induced inflammation in the respiratory tract (Mishra et al., 2008). However, exposure to nicotine-containing aerosol upregulates genes associated with the production of reactive oxygen species (ROS) and epithelium differentiation (Moses et al., 2017). Inhaled nicotine is suspected to induce airway remodeling by inhibiting complex III in the mitochondrial oxidative phosphorylation and leading to inhibition of myofibroblast differentiation (Javed et al., 2017; Lei et al., 2017). Further, inhaled nicotine disrupts the epithelium by inducing bronchial epithelial cell apoptosis (Bodas et al., 2016). Thus, nicotine has shown to have proinflammatory, anti-inflammatory, and potentially immunosuppressive effects on the respiratory tract.

### ENDS Flavorings Disturb the Professional Cells Phagocytic Activity, Antimicrobial Production, and Mucociliary Clearance in the Respiratory Tract

**FIGURE 1:**
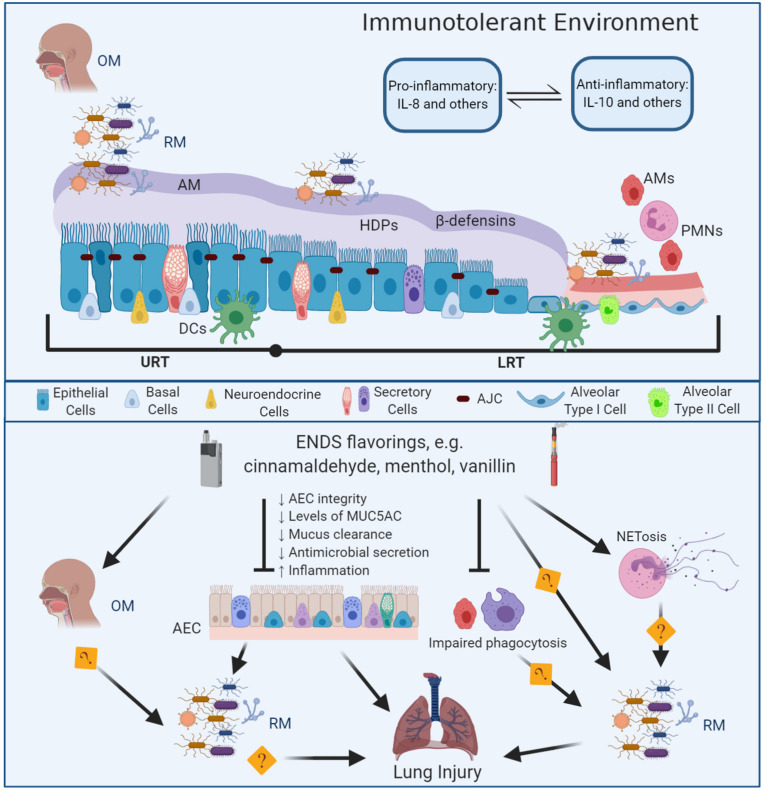
Mechanisms of E-liquid flavorings effects on the lung innate immunity and the respiratory microbiota in lung injury. The respiratory tract is lined by a pseudostratified columnar ciliated epithelium (nasal cavity through the bronchi) transitioning to cuboidal on the bronchioles and squamous on the alveoli (AEC) (Khan and Lynch, 2020). The apical junctional complexes (AJC), including tight and adherens junctions (Yuksel and Turkeli, 2017), maintain the airways epithelial integrity and contribute to prevent contact between the respiratory microbiota and the phagocytic cells therefore, providing an immunotolerant environment an immunotolerant environment (Mathieu et al., 2018). At the AJC, the tight junctions regulate paracellular permeability and the adherens junctions provide cell-to-cell communication, while connecting to cellular cytoskeletal proteins (Hartsock and Nelson, 2008; Rezaee and Georas, 2014). The airway mucus (AM) constitutes the water-based apical layer of the airway surface liquid and the periciliary layer is the second sol-based layer that bathes the epithelium (Widdicombe, 2002a). The respiratory microbiota (RM) resembles the oral microbiota (OM). Members of the RM relies on the airway mucus to access nutrients including mucins (MUC5AC and MUC5B) and avoid contact with the epithelium, professional antigen presenter cells (DCs) and phagocytic cells such as alveolar macrophages (AMs) and neutrophils (PMNs). The airway epithelium secretes β-defensins and other host defense peptides and proteins (HDPs). In addition to the airway’s epithelium, the innate immune response within the respiratory tract involves phagocytic cells including dendritic cells, alveolar macrophages, neutrophils (Agostini et al., 1993; Allie and Randall, 2017); eosinophils (Mesnil et al., 2016); innate lymphoid cells and natural killer cells (Cong and Wei, 2019). The airway surface liquid represents an ecological niche for the respiratory microbiota, provides a source of specialized nutrients, and plays a role in immunomodulation by regulating the interaction between the respiratory microbiota and the immune system (Hoskins and Zamcheck, 1968; Ouwerkerk et al., 2013; Sommariva et al., 2020). ENDS flavorings such as cinnamaldehyde, menthol, or vanillin can disrupt microbial clearance in the respiratory tract by disrupting the AEC integrity and decreasing the levels of mucus, decreasing mucus clearance and antimicrobial secretion, and eliciting and inflammatory response, eventually producing lung injury. ENDS flavorings also impair the phagocytic activity of AMs and PMNs. Further research is needed to understand how changes induced by ENDS flavorings in the OM, alveolar macrophages phagocytic activity, NETosis, induced by ENDS flavors, may alter de RM eventually producing lung injury.

ENDS flavoring chemicals play a major role on the cytotoxic effects of e-liquids and e-liquid aerosols (Allen et al., 2016; Gerloff et al., 2017). Exposure to ENDS flavorings results in abnormal activation of the lung epithelial cells and β-defensins and secretion of interleukin-8 (IL-8). Shen et al. (2016) showed that exposure to ENDS aerosol might also increase the gene expression of β-defensins in human bronchial epithelial cells (HBE) suggesting a proinflammatory response. Gerloff et al. (2017) showed ENDS flavorings might disrupt the epithelium integrity and impair the barrier function using an HBE *in vitro* model. Moreover, Gerloff et al. (2017) showed specific ENDS flavorings could stimulate a proinflammatory response by increasing the secretion of IL-8 in HBE and fibroblasts, which in turn will have a chemotactic effect on neutrophils. Clapp et al. (2017) determined that e-liquids containing cinnamaldehyde inhibit the immune function of the epithelium, neutrophils and natural killer cells in a dose-response manner, causing dysfunction of the macrophage phagocytic activity (Scott et al., 2018) and impaired bactericidal effect (Hwang et al., 2016). Recent data suggest that exposure to ENDS extract impairs neutrophil chemotaxis, inhibits ROS production and subsequently the neutrophil extracellular trap formation [i.e., an abnormal activation of the neutrophilic response (NETosis)] (Hickman et al., 2019; Corriden et al., 2020; Figure 1).

Additionally, Clapp et al. showed that exposure to cinnamaldehyde-containing ENDS might decrease the mucociliary escalator motility by reducing bioenergetic activity (Clapp et al., 2019) and may increase the levels of MUC5AC (Reidel et al., 2018). Preliminary reports have described the antimicrobial properties of menthol against *Staphylococcus aureus*, *Streptococcus epidermidis*, and *Micrococcus luteus* (Odeyemi and Oluwajoba, 2011) and members of the cigarette microbiota (Chopyk et al., 2017). ENDS menthol flavorings disrupt innate immunity and might be associated with allergies and asthma through activation of transient receptor potential ankyrin 1 (TRAP1). TRAP1 receptors (activated by menthol) might be involved in the chronic inflammatory response by stimulating the calcium-mediated secretion of substance P (neurogenic inflammation) in models of colitis (Guimaraes and Jordt, 2006; Engel et al., 2011), arthritis (Fernandes et al., 2011), and asthma (Tränkner et al., 2014; Deering-Rice et al., 2015; Yang and Li, 2016). Though the evidence base supporting harmful effects of the inhalation of flavoring chemicals on the respiratory innate immunity has strengthened, specific mechanisms or biomarkers describing short-and long-term effects are currently unknown. The information on the effects of ENDS flavoring chemicals and their potential effects on the lung innate immunity and the respiratory microbiota is still very limited. Future efforts have to focus on development of appropriate models to study these interactions accurately and allow discrimination between changes in the airways microbiota and the innate immunity as a consequence of exposure to flavorings from ENDS or as a mitigating effect. Further, it is not known whether nicotine, or humectant PG/VG and/or flavorings can alter the respiratory microbiodata leading to immune-inflammatory responses.

## The Respiratory Microbiota Might Modulate Innate Immune Responses in Vaping-Induced Lung Dysfunction

Little is known about the short- and long-term effects of ENDS on the respiratory microbiota, their impact on the innate immune response and their link to pulmonary health and disease. Preliminary studies have expanded our understanding of the relationship between the homeostasis of lung innate immunity and the immunomodulatory effect of the host-microbiota interaction. Existing and emerging evidence from *in vitro*, animal, and human studies are increasingly showing a link between the pathophysiology of respiratory disease and the exposure to ENDS aerosols (Chun et al., 2017; Gerloff et al., 2017; McConnell et al., 2017; Kaur et al., 2018; Chand et al., 2019; Gotts et al., 2019). Relatedly, alterations of the normal respiratory microbiota have been associated with COPD (Sze and Morris, 2016; Wang et al., 2017), asthma and atopy (Stiemsma and Turvey, 2017), and cystic fibrosis complications (Caverly et al., 2019), which are strongly associated with smoking and potentially with ENDS use. In addition, evidence is showing that the host-respiratory microbiota interaction plays a key role on inflammation, development and exacerbations in COPD, the risk and severity of asthma, and lung cancer.

Few studies have focused on the relationship between the respiratory microbiota and the immune response to air pollutants and toxicants in the respiratory tract. Fuochi et al. (2020) recently reported that nicotine and flavors from ENDS exposure enhanced the antibacterial effects of PG and VG, suggesting that ENDS exposure might potentially induce disturbances of the respiratory microbiota. As the respiratory microbiota is influenced by the bacterial membership of the oral and/or nasal microbiota, the effects of ENDS in the latter might relate to changes in the URT and LRT. Exposure to pollutants and toxicants might impact the respiratory microbiota and, therefore, the lung immunity. The respiratory microbiota composition may affect the innate immune responses by inducing defective phagocytic activity by alveolar macrophages and neutrophils, stimulating the secretion of immunoglobulin A and antimicrobials, increasing the mucus production, and disrupting the mucociliary activity. All these mechanisms have been associated with the pathophysiology of COPD, asthma, cystic fibrosis, and other respiratory conditions in relationship to smoking. Likewise, recent evidence suggests ENDS flavorings produce DNA damage in the airway epithelium, induce inflammatory response by increasing interleukin-8 (IL-8) or prostaglandin E2 (PGE2), and elicit oxidative stress (Lerner et al., 2015; Muthumalage et al., 2017; Kaur et al., 2018). Thus, it is biologically plausible that the exposure to ENDS flavorings can disrupt the respiratory microbiota contribution to homeostasis by altering its composition, which in consequence will impair the innate immune response in the respiratory system, increasing susceptibility to infective and inflammatory lung disease. Further research is needed to understand the effects of ENDS flavorings on the respiratory microbiota and its relationship to lung injury.

## Conclusion and Future Directions

The recent EVALI outbreak in the US, coinciding with a surge in youth vaping, generated a movement in the research community to establish research priorities and address challenges to support the Food and Drug Administration and World Health Organization efforts to regulate and control ENDS (Crotty Alexander et al., 2020). Elucidating the potential immunotoxicity of ENDS flavorings is necessary to inform regulation of e-liquid manufacturing and to educate the public and health professional community about ENDS safety. The immune homeostasis in the respiratory tract is maintained through a well-coordinated interplay among the respiratory microbiota, the airway epithelium, and the other elements of the innate immunity. ENDS flavorings induce abnormal activation of the lung epithelial cells and β-defensins, impaired macrophage phagocytic activity, increased levels of MUC5AC and NETosis. Growing evidence indicates that the respiratory microbiota might mediate the response to inhaled toxicants, however, many questions remain unanswered. Areas of future research include: (1) understanding the short- and long-term effects of ENDS flavorings in the dynamics of a healthy respiratory microbiota-host interaction and involvement of base PG/VG and/or nicotine; (2) elucidating the mechanisms and pathways of the respiratory microbiota-host interaction involved on the pathogenesis of flavor-vaping induced lung dysfunction; (3) determining whether the respiratory microbiota is a mediator or an initiator in flavor-vaping induced lung injury, thereby leading to lung diseases and their exacerbations; and, (4) developing *in vitro* and *in vivo* models for the realistic evaluation of the respiratory microbiota-lung innate immune response in the context of immunotoxicity studies. Further research on the pathogenesis of flavor-vaping induced lung injury must consider the respiratory microbiota as a potential mediator of the immune and the inflammatory response after exposure to toxicants.

## Author Contributions

ZQT, DJO, and IR initiated the idea for writing the manuscript, reviewed the literature, and edited the manuscript. ZQT drafted the manuscript. ZQT, DJO, IR, DC, DL, and SG revised, edited, and approved the final version of the manuscript.

## Conflict of Interest

The authors declare that the research was conducted in the absence of any commercial or financial relationships that could be construed as a potential conflict of interest.
